# Does Therapeutic Zinc Level of Supplementation for Diminutions of Acute Diarrheal Morbidity Varied in Public and Private Health Institutions in Ethiopia, Data from EDHS 2016?

**DOI:** 10.1155/2022/9975917

**Published:** 2022-08-05

**Authors:** Fassikaw Kebede, Merkineh Markos

**Affiliations:** Department of Epidemiology & Biostatics, Woldia University, College of Health Sciences, Woldia, Ethiopia 2022

## Abstract

**Background:**

Supplementation of zinc is a therapeutic medication for under-five children diminution incidence, severity, duration, and intensity of acute diarrhea morbidity. Nevertheless, levels of therapeutic zinc supplementation varied across public and private health institutions in Ethiopia. Thus, this study was aimed at estimating the levels of therapeutic zinc supplementation and factors associated for intent to be utilized among caregivers with their dyads, data from Ethiopia Demographic and Health Survey (EDHS 2016).

**Methods:**

The data used were from a secondary analysis of the Ethiopia Demographic and Health Survey in 2016 (EDHS). Overall, 1090 under-five children with acute diarrheal cases of two weeks before the EDHS 2016 were included. After cleaning, editing, and coding variables, the result was presented with frequency, tables, and graphs. Bivariable and multivariable logistic regression was conducted to identify and determine factors associated after zinc is prescribed for utilizations by caregivers.

**Result:**

The mean (±SD) age of participant children was found to be 36.4(±7.07) month. The overall levels of therapeutic zinc supplementation were 38.7% (95% CI: 35.8, 41.6) in public (29.08%) and private 138 (12.66%), respectively. The prescribed therapeutic zinc was influenced for utilization through maternal educational status (AOR = 2.55; 95% CI: 1.95, 3.47; *P* = 0.001), availability of health insurance (AOR = 10.7; 95% CI: 7.2, 16; *P* = 0.001), media exposure status (AOR = 2.1; 95% CI: 1.7, 3.6; *P* = 0.001).

**Conclusion:**

More than twofold time therapeutic zinc was prescribed in public than in private health institutions. Health care workers should be encouraged both in public and private health institutions for zinc prescription.

## 1. Introduction

Worldwide, diarrhea is the leading cause of hospitalization and responsible for 17.5–21% of all deaths for under-five children [1–3]. Even though with availability of simple and effective treatment for diarrhea, approximately 480,000 under-five children yearly died due to diarrhea [4]. Zinc supplementation in children decreases the incidence, duration, and severity of diarrhea duration 19.7% and mortality 23%, following supplementation [4, 5]. Despite the administration of zinc is one of the most cost-effective ways of preventing death among children from diarrhea, nearly all low- and middle-income countries have the policy to use zinc for diarrhea treatment [6].

The prescription and intent to give therapeutic zinc for diminutions of acute diarrhea varied across the world, 49% in Bangladesh, 18% in Tanzania, 10% in Nigeria, 15% in Sudan, and 21.54% in Ethiopia [1]. Of the East African countries, Uganda had the highest (40.51%) and Comoros was the lowest (0.44%) ever reported countries [1, 4]. The recommended dosage of therapeutic zinc for the treatment of acute diarrhea is 20 mg per day for above six and 10 mg per day for under six months of children until 10–14 days were prescribed [1]. The low level of zinc in serum is estimated to be responsible for 21% of worldwide deaths for under-five age children [7]. Particularly, it can demote the number and functioning of neutrophils and natural killer cells [8]. This may account for the decrease in the concentrations of cytokines such as IFN-*γ* and IL-2 that are produced by Th1 cells [6, 9], which are responsible for normal physiological functions of cell function and intracellular and extracellular functions beyond infection prevention.

Even if the World Health Organization (WHO) and United Nations Children Fund (UNICEF) highly recommended the inclusion of zinc for acute diarrhea, however the prescription of therapeutic zinc was varied across private and public institutions for cases [1, 4]. Accordingly, media exposure and carrier status of professionals determined zinc in each health institution [10]. Accordingly, the World Health Organization (WHO) and United Nations Children Fund (UNICEF) recommended the inclusion of zinc in the treatment of childhood diarrhea [11]. However, evidence is limited on zinc prescription practice, perceived cost, and willingness to pay for and related factors both in health professional and health care providers [12, 13]. Therefore, this investigation was done to assess levels of therapeutic zinc supplementation prescription practice and associated factors for acute diarrheal morbidity in both private and public health institution further analysis from data of EDHS 2016.

## 2. Methods and Setting

The data set used for this study had been retrieved from the 2016 EDHS data set which is conducted at national levels from January 18, 2016, to June 27, 2016, all over the country. This national-level survey was accompanied by a population-level cross-sectional study, and it is available at https://dhsprogram.com/data/available-datasets.cfm [4].

### 2.1. Study Population and Sampling Procedures

The 2016 Ethiopia Demographic and Health Survey was selected and accompanied in two stages. In the first stage, 202 clusters in urban areas and 443 clusters in rural areas were randomly selected by using 84,915 prepared enumeration areas since the 2007 population-housing census-sampling frame. In the second cluster stage, 28 households were selected after the household listing was carried out per cluster. Then, a total weighted sample of 1228 living under-five children who was diarrhea within 2 weeks was selected for interview.

### 2.2. Outcome Ascertainment

Outcome variables for this research are prescriptions of zinc acetate (yes/no) for cases presenting with acute diarrhea both in public and private health institutions.

### 2.3. Data Processing and Analysis

After we had formally extracted the full data set from EDHS 2016 from the National Central Static agency (CSC), we continually coded, edited, and categorized it to suit further analysis using STATA (SE) version R-14. Descriptive statistics like the Pearson chi-square test were used to check the existence of a significant difference between private and public health institutions in zinc supplementation for cases. Bivariable and multivariable logistic regression was used for the final analysis. Categorical variables with *P* value of *<* 0.25 during bivariable analysis were candidate transferred for multivariable logistic regression model. A variable on adjusted odd ratio (AOR) with a 95% CI was taken as a significant association at *P* value *<* 0.05 and declared as statistically significant factor for therapeutic zinc utilizations.

## 3. Result

### 3.1. Sociodemographic Characteristics of Respondents

**Figure 1:**
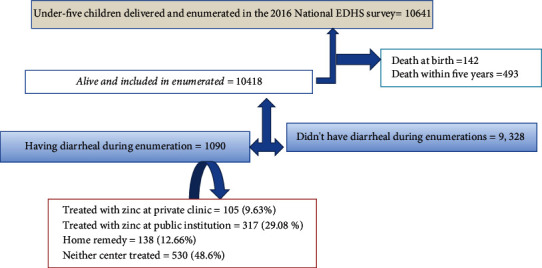
Sampling procedure of therapeutic zinc utilization for diminutions of acute diarrhea morbidity among under-five children in Ethiopia.

A total weighted sample of 1090 under-five age children paired with their caregivers was included in this analysis as acute cases presenting. Of the 10641 birth children, 142 (1.34%) died at birth. A majority (90.73%) of mothers got married. The overall mean (±SD) age of the study participants was 36.4(±7.07) years (Figure 1).

The majority 560 (51.38%) of the children were male in gender followed by female 530 (48.62%). The smallest proportions of 186 (17.06%) of the children were from rural, but majority (62.66%) of the caregivers had a good practice of hand washing. Regarding educational status, the majority of (59.7%) caregiver/mothers had no, and 90.73% were married. Moreover, 64.8% of mothers were breastfeeding, whereas 64.5% of caregivers had ≥5 families in a house (Table 1).

### 3.2. Levels of Therapeutic Zinc Supplementation Both in Public and Private Institutions for Presented Cases and Intended to Utilization through Caregivers

The overall levels of therapeutic zinc supplementations for diminutions of acute diarrhea episodes both in public and private institutions were determined 38.7% (95% CI: 35.86, 41.64) with a marked significant difference among categorical variables. The zinc supplementation in public health institution was found to be 29.08% followed with 138 (12.66%) in private health institutions. Moreover, caregivers of under-five children having health insurance and treated at public health institutes have a higher probability of utilizing therapeutic zinc for diminution of acute diarrheal morbidity with a significant difference (chi2(1) = 205.95; Pr = 0.001) from that of no health insurance (chi2(1) = 5.92; Pr = 0.062) (Table 2).

**Figure 2:**
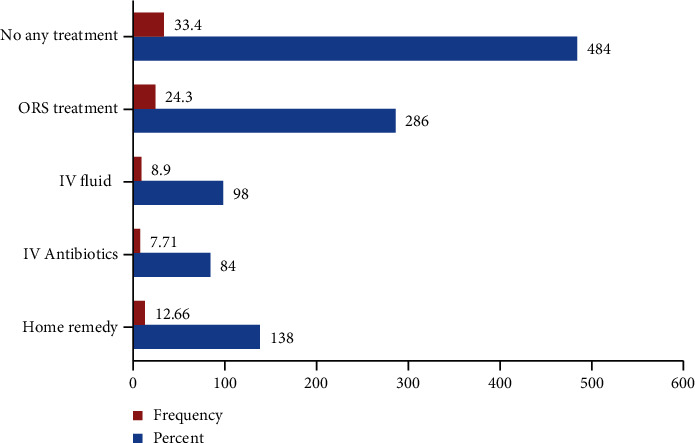
Types of medication given for diminutions of acute diarrheal morbidity for care seeking under-five children.

Mothers (caregivers) of under-five children rich in wealth index and treated at private health institution had a higher rate of zinc utilization for diminution of acute diarrheal episode (chi2 (1) = 64.69; Pr = 0.001; *P* = 1.265) as compared with those of poor wealth index. In addition, 286 (24.3%), 98 (8.9%), and 84 (7.71%) acute diarrheal cases were treated with ORS, IV fluids, and IV antibiotics as medication both in public and private health institutions, respectively (Figure 2).

### 3.3. Factors Determined Levels of Therapeutic Zinc Utilizations

As depicted in Table 3, bivariable logistic regression analysis was running, 17 variables were run, and subsequently, 15 variables were transferred into multivariable logistic regression with *P* value < 0.25 criteria of regression. In a final model of multivariable analysis, three variables were found to be significantly associated with zinc utilization after prescription.

The odds of supplemented therapeutic zinc utilization for diminutions of acute diarrheal morbidity among under-five children were 2.6 (AOR = 2; 95% CI: 1.9, 3.5; *P* = 0.001) times more likely higher among caregivers having formal education as compared with their counterparts. Moreover, the odds of having health insurance were 10 (AOR = 10.7; 95% CI: 7.2, 16.0; *P* = 0.001) times more likely to use therapeutic zinc after prescriptions as compared with caregivers who have no health insurance. Likewise, having media exposure (TV and radio) on prescribed therapeutic zinc utilization was 2 (AOR = 2.6; 95% CI: 1.7, 3.6; *P* = 0.001) times more likely to increase intention to use the prescribed zinc for diminution of acute diarrheal morbidity as compared with no media exposure among caregivers (Table 3).

## 4. Discussion

The overall therapeutic zinc supplementation for diminutions of acute diarrheal was found to be 37.7%. This finding is higher than the finding of the systemic review pooled report of systemic review 9% [14], USA 12% [15], and secondary data analysis of (DHS) East African countries 21.54 [1]. Conversely, this is a lower report than the finding in developing countries 45% [2]. This revealed that the magnitude is too far below with global recommendation of therapeutic zinc utilization [4, 16], and much effort is needed to increase the utilization due to the diminution of acute diarrheal morbidity and preventive bale causes of under-five mortality in the region. In some instances, there are knowledge differences among caregivers or mothers towards management and diminution of acute diarrhea following awareness creations for the implementation of a pilot projections of improving infant, young, and child feeding practices with the use of optimal micronutrient powders [2]. That means the implementation of the program might have an indirect role, with insufficient awareness resulting in higher rates of zinc utilization for infants and toddlers [16]. On the other hand, there is health information dissemination on how and when to use zinc after supplementation among cases of treatment institutions (public and private) [1, 4, 16]. The odds of supplemented therapeutic zinc utilization for diminutions of acute diarrheal morbidity among under-five children were 2.6 (AOR = 2; 95% CI: 1.9, 3.5; *P* = 0.001) times more likely higher among caregivers having formal education as compared with their counter group. This agreed with finding reported in Gondar [4] and reported in East African countries [1].

This might be because educated mothers might had knowledge regarding her child's health and are active to take her sick child to the nearest health facility. This in turn may create chance for mothers to get advice from health care providers and prescribed medication including zinc for their sick children. Generally, zinc supplementation reduces the incidence of diarrhea by 9% [17].

Another factor significantly associated with intensions to use therapeutic zinc utilization is in this report found to be the status of having health insurance. Moreover, the odds of having health insurance were 10 (AOR = 10.7; 95% CI: 7.2, 16.0; *P* = 0.001) times more likely to increase the use therapeutic zinc after prescriptions as compared with caregivers who have no health insurance. This is consistent with the previous finding in Gondar [4], secondary data analysis of (DHS) East African countries [1], and Egypt [18]. This might be rational that caregivers who have low wealth index are more likely to encounter budget constraints to afford the medication therapeutic zinc as a treatment for diarrhea for their children as compared to those who had a high wealth index. Likewise, having media exposure (TV and radio) on prescribed therapeutic zinc utilization was 2 (AOR = 2.6; 95% CI: 1.7, 3.6; *P* = 0.001) times more likely to increase intention to use the prescribed zinc for diminution of acute diarrheal morbidity as compared with no media exposure among caregivers. This is consistent with those reported in Jamshoro [12], India [9], sub-Saharan Africa, and Ghana [19]. Moreover, even in Ethiopia, reading newspapers and brochures and posting materials around private and public health institutions might have a great advantage for understanding zinc as an adjunct therapy for an important component for the management of diarrhea in under-five children.

## 5. Conclusion

More than twofold time therapeutic zinc was supplemented in public health institution than in private health institution. Health care workers shall be encouraged both in private and in public for zinc prescription intended for under-five cases presented with acute diarrheal morbidity. Health care workers should be encouraged both in public and private health institutions for zinc prescription.

## Figures and Tables

**Table 1:** Baseline sociodemographic and clinical characteristics of the study participants.

Variables	Categories	Weighted frequency	Percent
Sex of children	Male	560	51.38
	Female	530	48.62
Caregivers age group	15-19	2	0.18
	20-24	26	2.39
	25-29	185	16.97
	30-34	178	16.33
	35-39	267	24.50
	40-44	251	23.03
	≥45	181	16.61
Marital status	Married	989	90.73
	Unmarried	101	9.3
Residents	Urbane	186	17.06
	Rural	904	82.94
Regions	Large central	988	90.2
	Metropolitan	45	4.13
	Small peripheral	57	5.23
Educational status	Had no formal education	439	40.3
	Have formal education	651	59.7
Types of toilet	Modern	554	50.83
	Traditional	494	45.32
	Shared	15	1.38
	Not having at all	27	2.48
Having electricity	Yes	862	79.08
	No	228	20.92
Washing hand before feeding	Yes	683	62.66
	No	407	37.34
Wealth index	Poorest	422	38.72
	Poorer	139	12.75
	Middle	134	12.29
	Richer	159	14.59
	Richest	236	21.65
Health insurance	Yes	264	24.22
	No	826	75.78
Breastfeeding status	Yes	383	35.14
	No	707	64.8
TV in house	Yes	147	13.49
	No	943	86.51
Radio in house	Yes	243	22.29
	No	847	77.7
Wanted last children	Yes	685	62.8
	No	405	37.2
Levels of anemia	Anemic	160	14.7
	No anemic	930	85.3
Maternal occupations	No working	630	57.8
	Working	460	42.2
Family size	<5	387	35.5
	≥5	703	64.5

**Table 2:** Levels of zinc supplementation in public and private health institutions for diminutions of acute diarrhea cases on selected sociodemographic variables.

Variable	Categories	Therapeutic zinc supplementation status in public and private institutions							
		Public institution				Private institution			
		Given	Not given	Chi2(1)	*P* < value	Given	Not given	Chi2(1)	*P* < value
Sex	Male	165	395	1.486	0.223	53	507	1.03	0.8
	Female	142	388			52	478		
Resident	Urban	58	128	1.265	0.62	57	129	4.06	0.049
	Rural	353	551			260	644		
Health insurance	Had	215	49	463.0	0.001^∗^	23	744	1.3394	0.36
	Did not had	102	724			82	241		
Wealth index	Poor	214	473	3.8	0.05^∗^	99	583	64.7	0.001^∗^
	Rich	103	300			3	402		

**Table 3:** Bivariables intended to be used in therapeutic zinc utilization for the management of acute diarrhea morbidity among under-five children, EDHS 2016.

Variable	Categories	Therapeutic zinc supplementation		COR 95% CI	*P*-value
	Treatment center	Given	Not given		
Sex of child	Male	216 (19.8)	344 (31.5)	1.2 (1.12, 1.57)	0.034
	Female	9 (0.82)	335 (30.7)	1	
Resident	Urban	58 (5.3)	128 (11.7)	1	
	Rural	353 (32.3)	551 (50.5)	1.4 (1.12, 1.98)	0.028
Health insurance	Had	239 (21.9)	25 (2.3)	6.3(4.3, 9.4)	0.001
	No having	172 (15.7)	654 (60)	1	
Media exposer	Yes	47 (4.3)	364 (33.4)	1	
	No	100 (9.1)	579 (53.1)	1.39 (0.92, 1.9)	0.19
Electricity in house	Yes	56 (5.1)	355 (32.5)	2.1 (1.5, 2.99)	0.20
	No	172 (15.7)	507 (46.5)	1	
Hand washing	Yes	336 (30.8)	347 (31.8)	4.2 (3.2, 5.7)	0.01
	No	75 (6.8)	332 (30.4)	1	
Wealth index	Poor	199 (18.2)	223 (20.4)	1	
	Rich	81 (7.4)	58 (5.3)	0.4 (0.341, 0.58)	0.56
Marital status	Married	382 (35.0)	607 (55.6)	1.6 (0.99, 2.44)	0.18
	Unmarried	29 (2.6)	72 (6.03)	1	
Breastfeeding	Yes	149 (13.6)	234 (2.14)	1.1 (0.8, 1.39)	0.57
	No	262 (24.0)	445 (40..8)	1	
ORS given for cases	Yes	226 (20.7)	144 (13.2)	4.53 (3.4, 5.93)	0.001
	No	185 (16.9)	535 (49.0)	1	
Types of toilet used	Modern	197 (18.0)	357 (32.7)	0.31 (0.19, 0.479	0.03
	Traditional	203 (18.6)	291 (26.6)	1	
IV fluid	Given	72 (6.6)	653 (59.9)	5.3 (3.34, 8.51)	0.01
	Not given	26 (2.3)	339 (31.1)	1	
Home remedy as Rx	Given	5 (4.5)	406 (37.2)	1	
	Not given	133 (12.2)	546 (50.0)	19.7 (10.02, 48.7)	0.001
Educational status	Had formal education	359 (32.9)	257 (23.5)	11.3 (8.1, 15.76	0.001
	Had no formal education	52 (4.7)	422 (38.7)	1	
Hemoglobin	Anemic	73 (6.6)	338 (31.0)	1.46 (1.04, 2.2)	0.023
	Not anemic	87 (7.9)	592 (54.2)	1	
Last children wanted	Yes	267 (24.5)	418 (38.4)	1.15 (0.89, 1.49)	0.026
	No	144 (13.2)	267 (24.9)	1	
Region	Large central	350 (32.1)	633 (58.7)	1	
	Small metropolitan	61 (5.5)	46 (4.2)	2.39 (1.6, 3.5)	0.001
Media exposure	Yes	106 (9.9)	305 (27.98)	2.9 (2.13, 3.95)	
	No	87 (9.7)	592 (54.3)	1	

## Data Availability

All data that support the findings of this study is available from the corresponding author upon request.

## Abbreviations

AHR: Adjusted hazard ratio
CHR: Crude hazard ratio
CI: Confidence interval
FMOH: Ethiopian Federal Ministry of Health
LTFU: Lost to follow-up
MUAC: Midupper arm circumference
SAM: Severe acute malnutrition
NGT: Nasogastric intubation for feeding
WFH: Weight for height
SD: Standard deviation.
